# Longitudinal Changes of PAI-1, MMP-2, and VEGF in Peritoneal Effluents and Their Associations with Peritoneal Small-Solute Transfer Rate in New Peritoneal Dialysis Patients

**DOI:** 10.1155/2019/2152584

**Published:** 2019-05-05

**Authors:** Na Hao, Terry Ting-Yu Chiou, Chien-Hsing Wu, Yang-Yang Lei, Pei-Ling Liang, Mei-Chen Chao, Hongtao Yang, Jin-Bor Chen

**Affiliations:** ^1^Division of Nephrology, First Teaching Hospital of Tianjin University of Traditional Chinese Medicine, 88 Chang Ling Rd, Xi Qing District, Tianjin, China; ^2^Division of Nephrology, Department of Internal Medicine, Kaohsiung Chang Gung Memorial Hospital, Chang Gung University College of Medicine, Chung Shan Medical University School of Medicine, Taiwan

## Abstract

Patients on peritoneal dialysis (PD) encounter peritoneal functional and structural alterations. It is still unknown whether levels of plasminogen activator inhibitor type 1 (PAI-1), matrix metalloproteinases- (MMP-) 2, and vascular endothelial growth factor (VEGF) exhibit dynamic changes in peritoneal effluents. The aim of the present study was to investigate the longitudinal changes in these biomarkers in PD patients and their association with peritoneal small-solute transfer rate (PSTR). This prospective, single-center cohort study included 70 new PD patients. The presence of PAI-1, MMP-2, and VEGF in peritoneal effluents was measured regularly after PD initiation. The association between those biomarkers and 4-hour effluent:plasma creatinine ratio (PSTR) was analyzed. Longitudinal follow-up showed a tendency for PAI-1 (*p* < 0.001) and VEGF (*p* = 0.04) to increase with the duration of PD. Both PSTR at baseline and PSTR at 2 years significantly associated with PAI-1, MMP-2, and VEGF levels at baseline. PSTR at 2 years also associated with the MMP-2 level at 6 months and PAI-1 level at baseline. The present study illustrated a positive association of PSTR with selected biomarkers in peritoneal effluents observed over a 2-year period.

## 1. Introduction

Peritoneal dialysis (PD) is one of the renal replacement therapies employed in end-stage renal disease. Most common PD solution is glucose-based in clinical practice. There have been previous reports that peritoneal injury inferred from glucose-based PD solution in long-term dwell could lead to peritoneal functional and structural changes [1–3]. The most common functional alteration during long-term PD is increased peritoneal small-solute transport rate (PSTR) [1]. In clinical practice, peritoneal equilibration test (PET) is commonly applied to examine the solute transport rate in PD. The prognostic value of PET and patient outcomes have been extensively examined in the prior studies [4–6]. Basic principle in PET is obtained under certain conditions of peritoneal fluid and creatinine and the ratio of glucose in the blood thus determine the type of patients with peritoneal transport. However, the D/P creatinine (Cr) value from a single test of the PET is not sufficiently predictive of peritoneal transport, and monitoring the time-course changes is necessary. However, PET is cumbersome for sampling measurement and is time-consuming. Considering these disadvantages, several biomarkers that can be measured in the blood or PD effluent have been reported to be complementary indicators of peritoneal injury during PD therapy.

Plasminogen activator inhibitor type 1 (PAI-1), which has a molecular weight of approximately 50 kD, is produced by various cell types, including endothelial cells and vascular smooth muscle cells [7, 8]. PAI-1 is one of the factors involved in fibrinolysis during PD. One report claimed that the behaviors of PAI-1 and tissue-type plasminogen activator (tPA) in PD dialysate were not dependent on dextrose levels in the dialysate [9]. Therefore, PAI-1 in PD effluent has been reported as a biomarker associated with peritoneal modification, especially fibrosis [10].

The gelatinase, matrix metalloproteinases (MMP)-2 (molecular weight 72 kDa), degrades gelatin, collagen type IV, fibronectin, laminin, proteoglycan, and elastin [2, 10]. MMP-2 has previously been reported as a marker of peritoneal injury, increased solute transport, and encapsulates peritoneal sclerosis development [2, 11].

Vascular endothelial growth factor (VEGF) is a broad term referring to five isoforms of homodimeric glycoprotein with high heparin affinity [12]. VEGF induces endothelial cell proliferation and plays a key role in normal and abnormal angiogenesis. The strongest stimuli for VEGF production appear to be from ischemia and hypoxia [13]. Evidence suggested that high glucose PD solutions increased VEGF expression in the peritoneal cells [14, 15].

We hypothesized that glucose-based PD solutions would affect peritoneal structure and function. These alterations could be expressed in terms of concentrations of a variety of biomarkers obtained from PD effluents. Our study aimed to examine the serial changes in the concentration of biomarkers in PD effluents since PD initiation and the association of these changes with PSTR. Three biomarkers, PAI-1, MMP-2, and VEGF, were selected for the present study.

## 2. Materials and Methods

### 2.1. Subjects

Adult new PD patients who had commenced PD therapy since 2014 in our PD unit were included in the study. The inclusion criteria were patients with new catheter implant and receiving PD therapy for more than three months, age ≥18 years, and stable clinical condition during observational period. The exclusion criteria were uncompleted clinical information, discontinuation of PD therapy within six months due to death, shifting to hemodialysis, kidney transplantation, or transfer to other hospitals, advanced liver disease, malignancy, and incidence of peritonitis during the study period. All subjects were observed from September 29, 2014, to April 26, 2018.

The informed consent was obtained from individual subjects before study commencement. This study was approved by the Committee on Human Research at the Kaohsiung Chang Gung Memorial Hospital (Document no. 102-5925B), and the study was conducted in accordance with the principles of the Declaration of Helsinki.

### 2.2. Laboratory Measurements

Blood parameters from hemogram and biochemistry tests were measured once monthly. Standard PETs were performed at the first month and repeated every six months after PD commencement. Residual renal function (RRF) was calculated as the arithmetic mean of 24 h urea nitrogen and creatinine clearance, which were measured one month after PD commencement and at six-month intervals thereafter. RRF was normalized to body surface area using the Du Bois formula and patient body weight [16]. The recorded data included patient demographics, diabetic history, drug history, PD dialysis adequacy, and peritoneal transport category.

Peritoneal effluents were collected at overnight for MMP2, VEGF, and PAI-1 measurements when PET was performed at the first month, sixth month, and twenty-fourth month. All measurements were performed using commercial ELISA kits (quantitative sandwich ELISA, R&D Systems, Minneapolis, MN, USA).

### 2.3. Outcome Measures

The clinical outcome measure was PSTR which was defined as 4-hour PD dialysate:plasma creatinine (D/P Cr) ratio measured at PET.

### 2.4. Statistical Analysis

The baseline characteristics of individuals were summarized as mean and standard deviation, frequency and percentage, or median and interquartile range, as appropriate. The variation of inflammation markers from baseline to 6 months and that of 4-hour D/P creatinine from baseline to 2 years were estimated using paired* t*-test. The correlation between 4-hour D/P creatinine and inflammation markers at different time points was determined using Pearson's correlation test. The associations between inflammation markers, covariates, and 4-hour D/P creatinine measured at two-year follow-up were evaluated using linear regression analysis. The univariate regression analysis determined the association of individual biomarker with each variable. In addition, the multivariate regression analysis examined the associations of VEGF, PAI-1, and MMP-2 concentrations measured at two time points after adjustment of covariates, including age, gender, DM, angiotensin converting enzyme inhibitor (ACEi)/angiotensin receptor blockade (ARB), statin, hemoglobin, albumin, and glucose.* P*-value less than 0.05 was considered as statistically significant. All analyses were performed using the Stata version 11.0. (StataCorp. 2009, Stata 11 Base Reference Manual, College Station, TX: Stata Press).

## 3. Results

### 3.1. Baseline Characteristics

A total of 70 new PD patients were enrolled. The mean age was 56.4 years. Gender distribution was equal. Forty-one patients were treated with continuous ambulatory peritoneal dialysis (CAPD), 21 with ambulatory peritoneal dialysis (APD), and eight with CAPD+APD. There were relatively low percentage (n=7, 10%) in using ACEi or ARB or statin (n=30, 42.9%) in the participants. Peritoneal transport at the baseline was categorized as follows: high transport, 20.3%; high average transport, 33.3%; low average transport, 31.9%; and low transport, 14.5% (Table 1).

### 3.2. Concentrations of Biomarkers in PD Effluents

4-hour D/P creatinine had increased significantly from baseline (0.69) to 2-years (0.70) (*p*=0.02). VEGF (21.6 pg/mL to 22.2 pg/mL,* p* = 0.04) and PAI-1 (0.6 ng/mL to 1.1 ng/mL,* p* < 0.001) concentrations increased significantly from baseline to after six-month therapy. In contrast, MMP2 concentrations were constant during the six-month period (Table 2).

### 3.3. Correlation between PD Effluent Biomarkers and 4-Hour D/P Cr at Different Time-Point Measurements

Pearson's correlation test showed significant correlation between PSTR at baseline with PAI-1, MMP-2, and VEGF concentrations at baseline and PSTR after two years with PAI-1, MMP-2, and VEGF concentrations at baseline and MMP-2 levels at six months (Table 3).

### 3.4. Association between PD Effluent Biomarkers, Covariates, and 4-Hour D/P Cr at 2-Year Follow-Up

In univariate analysis, peritoneal effluent levels of VEGF, PAI-1, and MMP2 at baseline showed significant association with 4-hour D/P Cr at two-year measurement. These correlations were absent in multivariate analysis except for the peritoneal effluent PAI-1 (*β* 0.36,* p* = 0.039). Multivariate analysis revealed significant association between peritoneal effluent MMP-2 at six-month and 4-hour D/P Cr at two years (*β* 0.69,* p* < 0.001). Multivariate analysis showed that covariates did not show significant associations with 4-hour D/P Cr at either baseline or at two years (Table 4).

## 4. Discussion

It is well known that glucose-based PD solutions could provoke peritoneal membrane injury [1, 2, 10, 17]. The peritoneal membrane alterations include fibrosis, neoangiogenesis, and sclerosis [10]. For the past few years, researchers have been examining the association between several biomarkers obtained from peritoneal effluents and alterations in peritoneal function. Three biomarkers which were commonly investigated are PAI-1, MMP-2, and VEGF. Effluent PAI-1 and MMP-2 represent biomarkers associated with peritoneal fibrosis and VEGF represents biomarker associated with neoangiogenesis [8, 10, 11, 14, 18–20]. In the present study, we examined the potential of these biomarkers in PD effluent in predicting long-term PSTR in new PD patients. We also observed dynamic changes in these biomarkers in new PD patients during six-month PD therapy. We observed that PAI-1 and VEGF concentrations in PD effluents increased significantly during six-month PD therapy; however, the increase in MMP-2 concentration was not significant. Meanwhile, PAI-1 at baseline and MMP-2 at sixth month demonstrated significant association with PSTR (represented by D/P Cr at 4 hours) at two years. Grossly, our results were in corroboration with previous studies [2, 10, 14]. The studies on effluent biomarkers' concentrations revealed local production triggered by PD solution dwell and increased concentrations with longer PD therapy. These changes in concentrations are involved with complicated physiological and pathological alternations in peritoneal membrane. It is well known that both MMP-2 and PAI-1 are involved in several pathological and physiological processes, such as angiogenesis, cell growth, invasion, and metastasis [21]. PAI-1 is considered to be a protector by inhibiting plasminogen in the fibrinolytic system at injury sites and may converse pro-MMPs, and, thereby, increased MMP-2 levels and enhanced extracellular matrix degradation [10]. Moreover, MMP-2 could be upregulated during epithelial-to-mesenchymal transition of mesothelial cells [22]. Peritoneal angiogenesis also required MMP-2 and PAI-1 for activation and resolution [23]. These functional and structural alterations in peritoneal membrane were expressed in terms of change in biomarker concentrations in PD effluents.

The formation of fibrin on peritoneal surface has been associated with the appearance of adhesions in PD patients. It is already known that mesothelial cells exhibit fibrinolytic activity associated with t-PA production. In study with abdominal surgery model, PAI-1 was found throughout the submesothelial tissue when inflammatory reaction occurred [24]. However, one previous study did not reveal strong relationship between peritoneal tissue plasminogen activator (t-PA)/PAI system and the functional characteristics of the peritoneal membrane [18]. The PAI-1 concentrations in PD effluent represent circadian rhythm of fibrinolysis [9]. The concentrations were progressively elevated with dwell time in PD solution [9]. In the present study, we measured PAI-1 concentrations in PD effluent after overnight dwell. The timing of measurement of dialysate D/P Cr ratio was in 4-hour PD solution dwell. This time-discrepancy in sampling measurements may be discordant to the results obtained in prior studies. This time effect also influenced correlation test between PAI-1 concentrations and dialysate D/P Cr ratio in the present study. In our study, we found that baseline (PD therapy for one month) PAI-1 in PD effluent was significantly associated with D/P Cr ratio at two-year interval; however, the correlation disappeared with PAI-1 concentration at six months. Similar situation was also encountered in the association test with MMP-2. We found that MMP-2 measured in PD effluent at sixth month was significant association with PSTR, but the association disappeared with the MMP-2 measured at baseline. Thus, further study with uniform sampling measurement is necessary in the future such that a real relationship between biomarkers' concentrations obtained from PD effluent and PSTR could be clearly demonstrated.

VEGF is a mediator of neoangiogenesis. In long-term PD therapy with glucose-based PD solution, VEGF is present due to local production in the peritoneal membrane [14, 25]. Evidence suggests that VEGF plays an important role in peritoneal transport. VEGF level in PD effluents was correlated with the mass transfer area coefficient (MTAC) creatinine value and glucose absorption value and was negatively correlated with the transcapillary ultrafiltration [14]. In another cross-sectional study on PD patients, plasma and dialysate VEGF concentrations increased in patients with high average and high transporters compared to low average transporters [3]. An association between PSTR and plasma and dialysate VEGF concentrations was also demonstrated [3]. However, genetic polymorphism in VEGF could impact longitudinal change of peritoneal transport. The AA genotype exhibited significantly higher increase in 24-hour D/P creatinine compared to other genotypes [26]. In the present study, we did not find a significant association between VEGF in PD effluent measured at baseline and six-month interval and PSTR at two-year interval. Owing to short genetic polymorphism examination, we admit that interpretation of our results needs to be validated. A further detailed study is indicated to elucidate these relationships.

Present study is subject to several limitations. First, timing for sampling was different from prior studies. Thus, there is a discrepancy in head-to-head comparison. Second, we did not measure plasma concentrations of selected biomarkers. Therefore, we assumed that these biomarkers were produced locally in peritoneal membrane based on results of previous studies. Third, we did not examine the impact of genetic polymorphism in individual biomarkers on peritoneal transport. Fourth, we did not examine the inflammatory status of individual new PD patients during the study period by inflammatory markers test, such as CRP and IL-6. Therefore, the impact of inflammation in peritoneal cavity upon changes in biomarker concentration in PD effluents cannot be examined. Finally, the sample size was relatively small and may influence the statistical analysis. Nevertheless, the strength in our study was that a longitudinal peritoneal solute transport over two years in new PD patients was recorded and we examined the association with biomarkers' concentrations in peritoneal effluents during the first six-month PD therapy.

## 5. Conclusions

Our study demonstrated that PAI-1 and MMP-2 in PD effluents may be used as biomarkers in new PD patients with respect to relevant peritoneal transport parameter. Further studies with larger study population and varying degrees of peritoneal conditions are required to validate the results of present study.

## Figures and Tables

**Table 1:** Baseline characteristics of peritoneal dialysis patients (n = 70).

Variables	Mean	SD
Age (years)	56.4	±13.4
PD vintage (months)	2.1	±1.5
Gender, Male (n, %)	35	50.0%
Diabetes mellitus (n, %)	33	47.1%
ACEi/ARB (n, %)	7	10.0%
Statin (n, %)	30	42.9%
Laboratory measurements		
hemoglobin(g/dL)	10.4	±1.5
albumin (g/dL)	3.6	±0.4
fasting glucose(mg/dl)	106	93-198
PET measurements		
Category		
high transport	14	20.3%
high average transport	23	33.3%
low average transport	22	31.9%
low transport	10	14.5%
Total KtV	2.1	±0.5
Residual Renal Ccr (ml/min/1.73m^2^)	34.9	14.4-53.6
Total Ccr (ml/min/1.73m^2^)	73.3	56.8-86.3
nPCR(g/day)	1.1	±0.3

Paired test *p*-value is estimated using paired *t*-test.

PD, peritoneal dialysis; ACEi, angiotensin converting enzyme inhibitor; ARB, angiotensin receptor blockade; VEGF, vascular endothelial growth factor; PAI-1, plasminogen activator inhibitor-1; MMP2, matrix metalloproteinases 2; D/P Cr, dialysate: plasma creatinine.

**Table 2:** Temporary trends of dialysate inflammatory markers (n = 70).

Variables		Mean	SD
Dialysate inflammatory markers			
VEGF(pg/ml) (Baseline)		21.6	19.1-25.4
VEGF(pg/ml) (6 months)		22.2	19.7-27.5
	Paired test *p*-value	0.04	
PAI-1(ng/ml) (Baseline)		0.6	0.3-1
PAI-1(ng/ml) (6 months)		1.1	0.8-1.6
	Paired test *p*-value	<0.001	
MMP-2(ng/ml) (Baseline)		32.0	21.3-46.4
MMP-2(ng/ml) (6 months)		32.9	23.3-46.6
	Paired test *p*-value	0.73	
Outcome			
Follow-up interval (year)		2.24	1.96-2.46
4-hour D/P Creatinine (Baseline)		0.69	0.57-0.79
4-hour D/P Creatinine (2 years)		0.70	0.64-0.78
	Paired test *p*-value	0.02	

Paired test *p*-value is estimated using paired *t*-test.

PD, peritoneal dialysis; ACEi, angiotensin converting enzyme inhibitor; ARB, angiotensin receptor blockade; VEGF, vascular endothelial growth factor; PAI-1, plasminogen activator inhibitor-1; MMP2, matrix metalloproteinases 2; D/P Cr, dialysate:plasma creatinine; PET, peritoneal equilibration test; Ccr, creatinine clearance rate; nPCR, normalized protein catabolic rate.

**Table 3:** Correlation between inflammation markers and 4-hour D/P creatinine at different time-point measurements.

Variables	4-hour D/P Creatinine		4-hour D/P Creatinine	
	(Baseline)		(2 years)	
	*r*	*P*	*r*	*P*
VEGF(pg/ml)				
Baseline	0.36	0.002	0.26	0.029
6 months	0.10	0.404	0.14	0.248
PAI-1(ng/ml)				
Baseline	0.40	< 0.001	0.34	0.004
6 months	0.34	0.005	0.20	0.110
MMP-2(ng/ml)				
Baseline	0.64	< 0.001	0.32	0.006
6 months	0.59	< 0.001	0.56	< 0.001

*P*-value estimated using Pearson's correlation test.

*r*: Pearson's correlation coefficient.

**Table 4:** Linear regression analysis for association between biomarkers, covariates, and 4-hour D/P creatinine measured at two-year follow-up.

Variables	Univariate		Multivariate	
	Beta	*P*	Beta	*P*
VEGF(pg/ml)				
Baseline	0.26	0.029	-0.09	0.604
6 months	0.14	0.248	-0.001	0.994
PAI-1(ng/ml)				
Baseline	0.34	0.004	0.36	0.039
6 months	0.2	0.11	-0.28	0.103
MMP-2(ng/ml)				
Baseline	0.32	0.006	-0.07	0.694
6 months	0.56	<0.001	0.69	<0.001
Covariates				
Age (years)	-0.06	0.600	-0.13	0.257
Gender, Male (n, %)	0.06	0.613	-0.06	0.611
Diabetes mellitus (n, %)	0.07	0.564	0.01	0.902
ACEi/ARB (n, %)	0.19	0.107	0.17	0.113
Statin (n, %)	-0.12	0.337	-0.04	0.739
Hemoglobin(g/dL)	-0.03	0.788	0.04	0.719
Albumin(g/dL)	-0.24	0.047	0.02	0.888
Fasting glucose(mg/dl)	0.19	0.128	0.06	0.611

Multivariate model includes VEGF, PAI-1, and MMP2 measurements at two time points and adjusted for covariates including age, gender, DM, ACEi/ARB, statin, hemoglobin, albumin, and glucose.

## Data Availability

The raw data used to support the findings of this study are available from the corresponding author upon request.

## Abbreviations

PD: Peritoneal dialysis
PAI-1: Plasminogen activator inhibitor-1
MMP2: Matrix metalloproteinases 2
VEGF: Vascular endothelial growth factor
PSTR: Peritoneal solute transport rate
PET: Peritoneal equilibration test
tPA: Tissue-type plasminogen activator
RRF: Residual renal function
D/P Cr: Dialysate plasma creatinine
ACEi: Angiotensin converting enzyme inhibitor
ARB: Angiotensin receptor blockade
CAPD: Continuous ambulatory peritoneal dialysis
APD: Automated peritoneal dialysis
CCPD: Continuous cyclic peritoneal dialysis
MTAC: Mass transfer area coefficient.
